# Polystyrene Oxygen Optodes Doped with Ir(III) and Pd(II) *meso*-Tetrakis(pentafluorophenyl)porphyrin Using an LED-Based High-Sensitivity Phosphorimeter

**DOI:** 10.3390/s18061953

**Published:** 2018-06-15

**Authors:** Alexandre F. De Moraes Filho, Pedro M. Gewehr, Joaquim M. Maia, Douglas R. Jakubiak

**Affiliations:** 1 Federal Institute of Education, Science and Technology of Paraná (IFPR), Curitiba 80230-150, Brazil; 2 Graduate Program in Electrical and Computer Engineering (CPGEI), Federal University of Technology-Paraná (UTFPR), Curitiba 80230-901, Brazil; gewehr@utfpr.edu.br (P.M.G.); joaquim@utfpr.edu.br (J.M.M.); 3 Department of Electronics (DAELN), Federal University of Technology-Paraná (UTFPR), Curitiba 80230-901, Brazil; jakubiak@utfpr.edu.br

**Keywords:** iridium porphyrin, IrTFPP-CO-Cl, oxygen optode, palladium porphyrin, PdTFPP, phosphorimeter, time-resolved phosphorimetry, time-domain lifetime

## Abstract

This paper presents a gaseous oxygen detection system based on time-resolved phosphorimetry (time-domain), which is used to investigate O${}_{2}$ optical transducers. The primary sensing elements were formed by incorporating iridium(III) and palladium(II) *meso*-tetrakis(pentafluorophenyl)porphyrin complexes (IrTFPP-CO-Cl and PdTFPP) in polystyrene (PS) solid matrices. Probe excitation was obtained using a violet light-emitting diode (LED) (low power), and the resulting phosphorescence was detected by a high-sensitivity compact photomultiplier tube. The detection system performance and the preparation of the transducers are presented along with their optical properties, phosphorescence lifetimes, calibration curves and photostability. The developed lifetime measuring system showed a good signal-to-noise ratio, and reliable results were obtained from the optodes, even when exposed to moderate levels of O${}_{2}$. The new IrTFPP-CO-Cl membranes exhibited room temperature phosphorescence and moderate sensitivity: <$\tau_{0}$>/<$\tau_{21\%}$> ratio of ≈6. A typically high degree of dynamic phosphorescence quenching was observed for the traditional indicator PdTFPP: <$\tau_{0}$>/<$\tau_{21\%}$> ratio of ≈36. Pulsed-source time-resolved phosphorimetry combined with a high-sensitivity photodetector can offer potential advantages such as: (i) major dynamic range, (ii) extended temporal resolution (Δτ/Δ[O${}_{2}$]) and (iii) high operational stability. IrTFPP-CO-Cl immobilized in polystyrene is a promising alternative for O${}_{2}$ detection, offering adequate photostability and potentially mid-range sensitivity over Pt(II) and Pd(II) metalloporphyrins.

## 1. Introduction

Metalloporphyrins are an important class of organic compounds that are used in several areas of research. Besides their well-known catalytic action and established use in O${}_{2}$ sensing, they have also permitted the design of photovoltaic cells [1] and organic light-emitting diodes (OLEDs) [2]. Specifically in the area of medicine, other applications utilize these compounds as biosensors [3,4], photosensitizers for photodynamic therapy and contrast agents (for magnetic resonance imaging) [5].

Pt(II) and Pd(II) metalloporphyrins exhibit strong phosphorescence, even at ambient temperature. Varying brightness (from moderate to intense), a red/near-infrared (NIR) emission and good photostability are some of the qualities that justify the widespread use of these compounds in O${}_{2}$ detection [6]. Even more interesting, their lifetimes in the absence of a quencher ($\tau_{0}$) are relatively long [3]. Platinum porphyrins (PtPs) have a high quantum yield (Φ) reaching 50% in some cases, with $\tau_{0}$ values in the tens of microseconds. The Φ values for palladium porphyrins (PdPs) are usually 2–3-times lower, but their lifetimes easily surpass hundreds of microseconds. As a result, numerous natural and synthetic metalloporphyrins have been explored, and the indicators PtTFPP and PdTFPP (as well as the transition metal complex Ru(dpp)${}_{3}$ 2${}^{+}$) are the most commonly-used luminophores in O${}_{2}$ sensors. The classic metalloporphyrins mentioned above are currently being gradually replaced by π-extended porphyrins (i.e., for applications that benefit from NIR emission of benzoporphyrin derivatives), such as Pd(II) *meso*-tetrakis(4-fluorophenyl)tetrabenzoporphyrin (PdTPTBPF) [3,7,8,9,10].

The most common principle by which optical O${}_{2}$ transducers operate is based on the dynamic quenching process, namely attenuation of the intensity and/or lifetime of their luminescence as a function of the energy transfer from their excited state to a quenching agent (such as oxygen in the ground state) [3,11,12]. The design of the oxygen optodes normally utilizes a strategy that can tailor the luminophore to the application, especially to minimize aggregation and/or self-quenching. One of the most widely-used methods is based on creating thin or thick films (membranes), obtained by immobilizing the indicator in O${}_{2}$-permeable organic polymers [7,12]. Transduction of these elements is made possible by a sensing system, which usually involves optoelectronic devices, optical components, circuits to acquire and process signals and subsequent processing of measurement [10,13]. Transduction methods based on lifetime detection minimize errors inherent to measuring intensities (steady-state) such as fluctuations in the optical excitation source, photo-oxidation of the indicator, light scattering and displacement of the sensing phase [14,15,16]. If detection involves direct measurement of decay times, extended lifetimes reduce the complexity of the devices, since registering and processing the signal in detail is more feasible [7,17]. Consequently, Pt(II) and Pd(II) metalloporphyrins are technically suitable for implementing systems based on detecting lifetimes [18].

The porphine molecule, an aromatic macrocycle from which porphyrins originate, allows a variety of modifications ranging from peripheral alteration (adding substituents to the β-pyrrolic and/or *meso* positions) to the inclusion of different metal cations in the center of the ring, resulting in a metalloporphyrin complex. These modifications alter the photophysical properties of the compound, its solubility (making it more hydrophobic or hydrophilic), improve its stability and/or add different features (e.g., affinity for biological molecules) [3,19,20]. Phenyl groups in *meso*-positions of the porphyrin ring increase the solubility of the complex in solvents and organic polymers. If the hydrogen atoms on the periphery are replaced with fluorine halogens, greater solubility can be expected, but more importantly, photostability is dramatically improved (electron withdrawing effect) [3,21,22]. In fact, PtTFPP and PdTFPP metalloporphyrins are highly compatible with nonpolar polymers (except silicones) and exhibit very good brightness upon excitation in the Soret band, but these compounds are preferred for their excellent resistance to photo-oxidation [3,7,10,23,24]. Metalation with iridium, for example, results in a six-coordinate complex whose axial ligands can be modulated to tune the properties of the molecule [7,25,26,27]. Iridium complexes of octaethylporphyrin (OEP) and meso-tetra(phenyl)tetrabenzoporphyrin (TPTBP) were investigated by Koren et al. [25]. Despite exhibiting potentially intermediate sensitivity over their platinum and palladium counterparts, they still constitute a relatively more recent, less-exploited category of metalloporphyrins [3,25].

Indicators with longer lifetimes, such as PdPs, offer great sensitivity and provide high resolution in detecting low concentrations of O${}_{2}$ [7,17]. However, when they are exposed to a moderate presence of the analyte, they suffer excessive attenuation of phosphorescence, which makes them unusable due to the reduced signal-to-noise ratio [7,28]. The strategy normally adopted, other than replacement of the indicator itself, consists of using low-permeability polymers (e.g., poly(styrene-acrylonitrile)), which restricts access to the O${}_{2}$ and extends the upper limit of detection, but causes slow response to gas exchanges [3]. This work presents a less common means of broadening the range of detection when these indicators are used: combining pulsed-source time-resolved phosphorimetry with a high-sensitivity photodetector [7]. This approach can offer higher resolution over less sensitive indicators (e.g., PdTFPP versus PtTFPP) [28], without over-compromising the dynamic response of the sensing phase. Another advantage of this method is high operational stability, due to the lower excitation energy applied. To investigate the operation and particularly the sensitivity of the instrument setup, two polystyrene optodes were developed and characterized: the first utilizes the traditional PdTFPP indicator, while the second uses the same porphyric base, which is metalated with Ir(III), offering an innovative alternative for O${}_{2}$ sensing.

## 2. Materials and Methods

### 2.1. Preparation of the Oxygen Optodes

**Figure 1:**
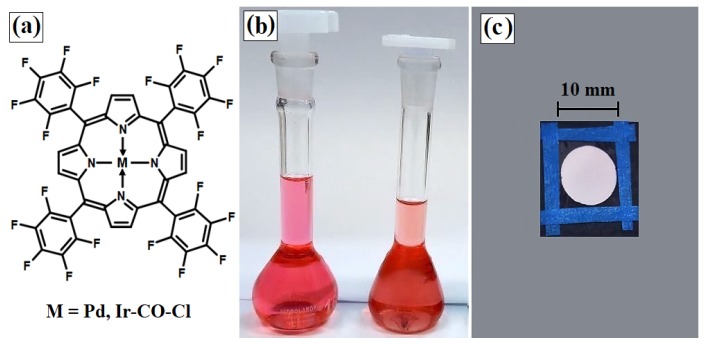
(**a**) Structural formula of the metalloporphyrins under study; (**b**) PdTFPP (left) and IrTFPP-CO-Cl (right) dissolved in CHCl${}_{3}$ and (**c**) polystyrene optode.

Initially, 90 mg of polystyrene (average molecular weight (Mw) = 260,000; PS U288, Unigel, São Paulo, Brazil) were dissolved in 4 mL of chloroform. In another flask containing 2.2 mL of toluene, 97 mg of the same polymer were mixed with 4 mg of poly(dimethylsiloxane-*co*-α-methyl styrene) (average Mw = 75,000, AB block copolymer , 20% dimethylsiloxane; Scientific Polymer Products Inc., Ontario, NY, USA). Then, 4 drops of the second solution were added to the first solution, along with 25 drops of toluene (using Pasteur pipettes). This process of “doping” the polymer solution improved the homogeneity of the membrane thickness (less roughness) and also eliminated any fogging during the drying process. To prepare the sensor cocktails with the compounds (Figure 1a), 0.5% w/w of IrTFPP-CO-Cl (CAS 1432321-43-1) and PdTFPP (CAS 72076-09-6) indicators (Frontier Scientific, Logan, UT, USA), which were previously dissolved in chloroform, were used (Figure 1b). These cocktails were stirred magnetically for approximately 3 h. Then, 8 drops (≈75 μL) of each solution were deposited on microscope slides using a pipetting process. After an hour, sensing membranes approximately 12 mm in diameter and 8 μm thick were formed and ready for use (the thickness was estimated by 3 measurements on different regions of the membrane with a micrometer; Mitutoyo 293-561-30, Kawasaki, Japan). Before their final use, the transducers were peeled from the glass (by immersion in deionized water), fixed in matte black cardboard (in 78 mm${}^{2}$ cutout holes) (Figure 1c) and subjected to heat treatment (24 hours at 70 ${}^{\circ }$C) [29].

### 2.2. Sensor Characterization

Absorption spectra for both the metalloporphyrins dissolved in chloroform and polystyrene optodes were measured using a 1601 UV-Vis spectrophotometer (Shimadzu, Kyoto, Japan). The emission spectra were obtained by excitation of the sensors in the Soret absorption band (violet LED). Emission spectra over the 200–1000 nm range were recorded on a QE65000 spectrometer (Ocean Optics, Dunedin, FL, USA). A home-built instrument (described in Section 2.2.1, i.e., “Instrument Setup”) was used to detect the phosphorescence lifetimes and to analyze the photostability of the sensor phases.

#### 2.2.1. Instrument Setup

**Figure 2:**
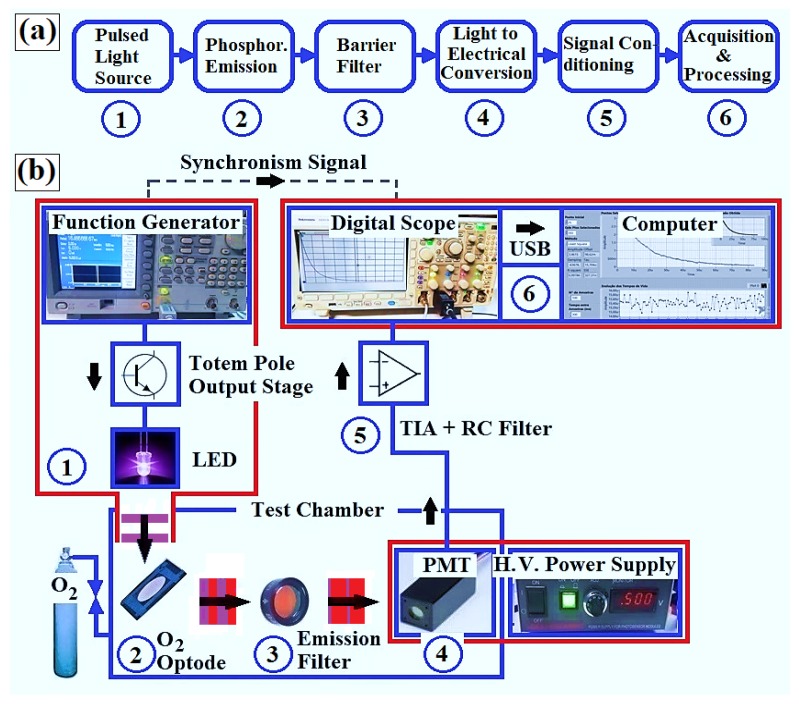
(**a**) Block diagram of the built instrument and (**b**) physical setup.

The transduction method applied is based on pulsed-source time-resolved phosphorimetry, as shown in Figure 2. This system has two main attributes: generation of a short-duration excitation beam and subsequent detection of the phosphorescent signal emitted by the primary sensor (free or natural response).

##### Pulsed Light Source (Figure 2b, No.1)

To excite the metalloporphyrins, a violet UV5TZ-405-30 LED (typical transmission power of 40 mW) (Bivar Inc., Irvine, CA, USA) was selected. Its relatively narrow emission band (full width at half maximum ≈20 nm), combined with the spectral separation between excitation and emission wavelengths of the indicators, does not require the use of an optical filter at the inlet of the system. In order to allow rapid switching of this device, its polarization is based on a totem-pole circuit designed using 2N3904 bipolar transistors (STMicroelectronics, Catania, Italy) (peak LED current pre-set at 18 mA). The excitation pulses are produced by an AFG3021C function generator (Tektronix, Beaverton, OR, USA), which provides some flexibility in the arrangement. In order to obtain a measurement uncertainty for lifetimes close to 1% when the optodes are exposed to ambient air [30], a pulse width of 5 μs/60 Hz (LED power dissipation ≈18 μW) was defined as an operating standard.

##### Test Chamber (Figure 2b, No.2)

**Figure 3:**
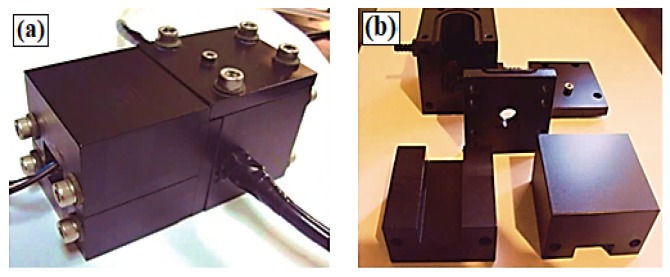
(**a**) Test chamber and (**b**) separated components.

The optical cell was built from an aluminum block (black, anodically oxidized), which was created to minimize optic paths, adjust the fit of the sensing membrane and hold the emission filter in relation to the photodetector (Figure 3). This chamber not only acts as a darkroom, but also provides electromagnetic shielding and strong mechanical protection for the photosensor module. The optode is inserted in the right-hand block through the top cover (Figure 3a). This is where gaseous exchanges occur, through two external connections. This same block also has additional holes to attach the excitation LED and a temperature sensor (PT100, Novus, Porto Alegre, Brazil). The distances from the LED to the center of the optode and from the center of the optode to the photosensor module window are 1.8 cm and 2.0 cm, respectively (this positioning allows full use of the O${}_{2}$ transducer’s working area). The left-hand block is used to encapsulate the photomultiplier tube (PMT). The interlock between the blocks is an 8 mm-thick plate, which contains a window for the optical filter (Figure 3b). The LED and PMT detection window are positioned at a 45${}^{\circ }$ angle to the sensor membrane (Figure 2b, No.2), thus reducing the capture of the excitation signal by the photodetector (which is necessary despite the use of the emission filter).

##### Emission Filter (Figure 2b, No.3)

To selectively detect phosphorescence, namely attenuating the light scattered from the LED, an 86-942 band-pass filter (Edmund SC., Barrington, NJ, USA) was positioned in front of the photodetector. This filter has a minimum of 90% transmittance at 675 nm, full width at half maximum (fwhm) = 50 nm and optical density of 4.

##### Photomultiplier Tube (Figure 2b, No.4)

Considering that sensitivity is a preponderant factor in adequately detecting phosphorescence, especially when the optodes are subjected to moderate concentrations of O${}_{2}$, an H10721-20 photosensor module (Hamamatsu, Hamamatsu, Japan) was selected. One specific feature of this device is its peak response at 630 nm (typical cathode radiant sensitivity of 78 mA/W), not the usual violet range, which substantially benefits the signal-to-noise ratio (SNR) of the system. The tube also has an 8 mm-diameter detection window, rapid response (0.57 ns) and typical dark current of 10 nA [31].

##### Conditioning of the Phosphorescent Signal (Figure 2b, No.5)

**Figure 4:**
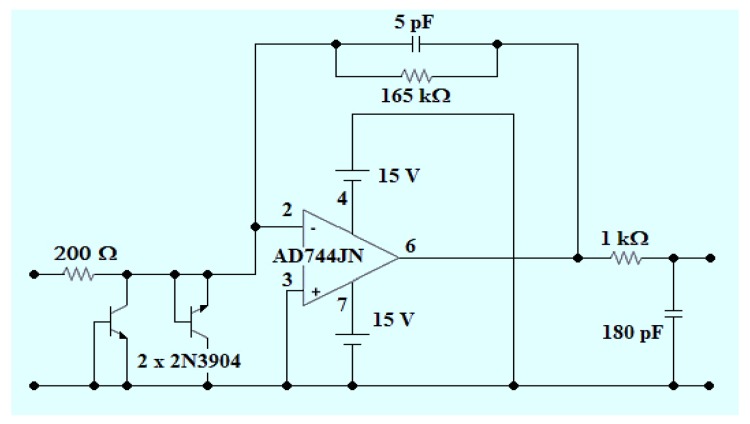
Signal conditioning circuit.

The electric current generated by the photosensor module is converted into voltage by a transimpedance amplifier (TIA) based on an AD744JNZ operational amplifier (Analog Devices, Norwood, MA, USA). This integrated circuit has a typical slew rate of 75 V/μs, a bandwidth of 13 MHz and voltage noise of 18 nV/1 kHz. The TIA was designed with a resistor-capacitor network of 165 kΩ and 5 pF, respectively (Figure 4). This network increases stability and improves the SNR of the amplifier, but creates a delay (time constant of 825 ns) in the response of the system [32], limiting phosphorescence lifetime detection to a minimum of 8 μs (about 10-times the time constant of the TIA, i.e., an error less than 0.5%; not considering the possibility of a potential deconvolution). For an additional increase in SNR, a first-order passive low-pass filter was also used.

##### Lifetime Acquisition and Processing (Figure 2b, No.6)

The voltage signal decay (corresponding to phosphorescence) is obtained and then recorded using an MDO3014 digital oscilloscope (100 MHz, 2.5 GS/s) (Tektronix, Beaverton, OR, USA). The oscilloscope not only allows visualization of signals corresponding to the polarization of the LED and phosphorescence decay, but also acquires and performs the analog-to-digital conversion of this decay, allowing adjustment of the discretization of the signal of interest (pre-fixed for 1000 points), as well as the selection of the desired number of averages (signal averaging). Thus, this instrument plays the role of an acquisition board, which limits the temporal resolution to its time base (horizontal scale). Furthermore, the acquisition signal is synchronized by the descending signal of the LED excitation pulse, guaranteeing repeatability of the detection cycle. For storage and/or processing of the experimental data, this device was connected to a standard-use laptop computer (Lenovo Model G50 Intel Core i5, Itu, Brazil) via a USB port. Decays were fit to lifetimes by employing a Levenberg–Marquardt nonlinear least-squares algorithm, using a program developed in LabVIEW (National Instruments 2015, Austin, TX, USA).

### 2.3. Lifetime and Photostability Measurements

In order to avoid the cross interference, temperature and pressure conditions were controlled (23 ${}^{\circ }$C and 300 Pa, respectively). For the calibration curves (static sensitivity of the sensors), different concentrations of O${}_{2}$ were used with nitrogen gas as an inert component (from gas cylinders with an absolute uncertainty <0.05%; Praxair, Araucária, Brazil). The respective phosphorescence lifetimes were obtained from 100 consecutive measurements, with each measurement corresponding to the average of 8 successive decays (SNR is proportional to the square root of the number of decays averaged) [33]. The acquisition interval of phosphorescence signal used to calculate lifetimes was considered valid between 5 μs (time delay after switching off LED to start the acquisition) [12,34], and five-times the time constant pre-measured. These decay curves were modeled assuming a double exponential decay behavior [35]:

(1) $$I\left(t\right)=\alpha_{1}.e^{\frac{-t}{\tau_{1}}}+\alpha_{2}.e^{\frac{-t}{\tau_{2}}},$$

where *I*(*t*): phosphorescence intensity during any instant of time *t*; $\alpha_{1}$ and $\alpha_{2}$: initial intensities for the exponential components; and $\tau_{1}$ and $\tau_{2}$: their respective phosphorescence lifetimes for a given [O${}_{2}$]. From the two components (longer and shorter lifetimes), the amplitude-weighted lifetime (<τ>) was calculated using the following equation [35]:

(2) $$<\tau >=\alpha_{1}.\tau_{1}+\alpha_{2}.\tau_{2}.$$

Alternatively, a monoexponential model was also used to facilitate lifetimes’ processing and later to obtain a two-point calibration curve of the practical instrument [30,36,37,38]:

(3) $$I\left(t\right)=I_{I}.e^{\frac{-t}{\tau }}+b,$$

where *I*(*t*): phosphorescence intensity during any instant of time *t*; *I${}_{I}$*: initial phosphorescence intensity; τ: phosphorescence lifetime for a given [O${}_{2}$]; and *b*: residual signal value (offset).

For photostability tests, the sensor membranes were reallocated in an external device before being exposed to more intense energy (which would certainly cause permanent damage to the photosensor module). The relative position between the LED and sensor membrane was identical to the mechanical configuration of the sample chamber, but instead of a pulsed signal, the LED was excited using a continuous 20 mA current. In this case, the changes resulting from phosphorescent decay in the presence of ambient air were verified.

## 3. Results and Discussion

### 3.1. Spectra Measurements

**Figure 5:**
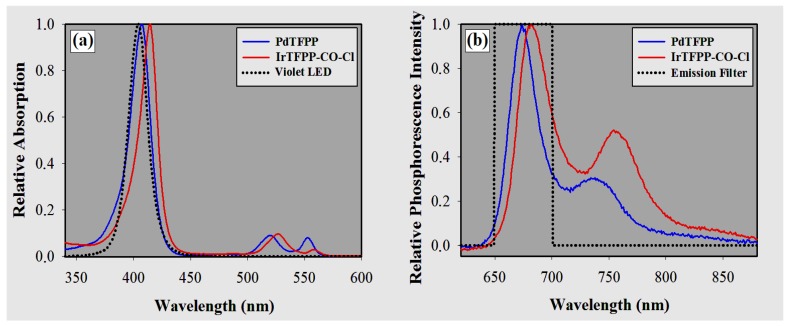
(**a**) Absorption spectra in chloroform and (**b**) phosphorescence spectra in polystyrene for PdTFPP and IrTFPP-CO-Cl at 23 ${}^{\circ }$C.

Table 1 presents the optical properties for the indicators and optodes under study. In general terms, immobilization in PS (compared to the chloroform solvent) caused a bathochromic shift of only 3 nm in the peaks of the Soret band (B band). Comparing to PdTFPP, the absorption and emission bands of iridium metalloporphyrin are bathochromically shifted by ≈7 nm (Figure 5). Furthermore, PdTFPP and Ir-TFPP-CO-Cl presented secondary emission peaks in the NIR range of about 30% and 50%, relative to their maximum peaks (Figure 5b).

### 3.2. Residual Excitation Light

To validate the choice of time delay used to start the acquisition of the phosphorescence decays, a polystyrene membrane was inserted into the test chamber without the indicator. This procedure allowed visualizing a monoexponential decay (scattered light at the polymer) with a maximum amplitude of 150 mV, which disappeared almost completely after approximately 4 μs. Thus, 5 μs was considered sufficient to eliminate any residual light from the optical source and was employed as time delay (this was necessary since the interference filter is not ideal and also the PMT is extremely sensitive). Besides that, the base noise from the system was below 1 mV peak-to-peak (considering promediation of signals), while the offset voltage was practically nil.

### 3.3. Sensitivity of the Oxygen Optodes

#### 3.3.1. Brightness

**Figure 6:**
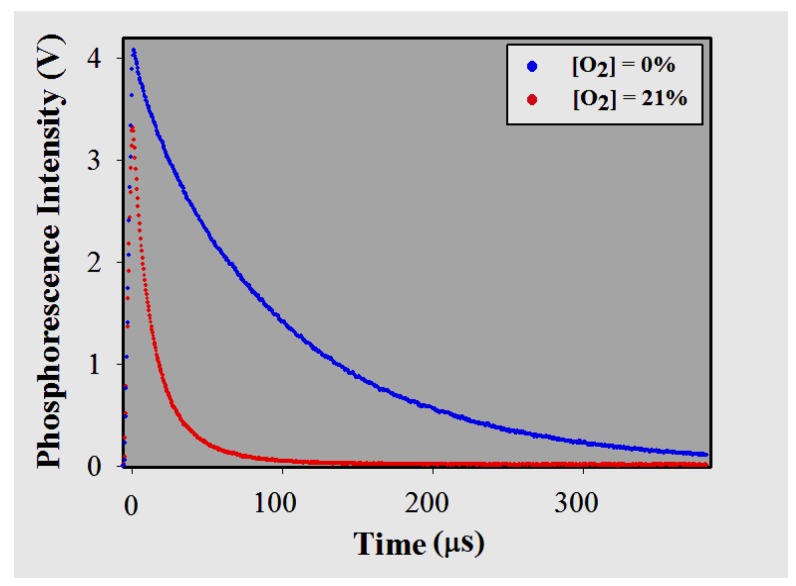
Phosphorescence decay for IrTFPP-CO-Cl immobilized in PS.

Compounds with significant luminescence quantum yield (Φ) alone do not guarantee sufficient light emission unless they conveniently absorb excitation energy. Since the molar absorption coefficients (ε) of the two metalloporphyrins exceed 180,000 M${}^{-1}$ cm${}^{-1}$, their brightness (defined as the product of ε and Φ) is very good upon excitation in the Soret band [3,7,39]. Figure 6 shows the phosphorescence decay profile for the membrane doped with IrTFPP-CO-Cl (gain over fixed photosensor module at its minimum value, 5000). In the absence of gaseous oxygen, its relative quantum yield ($\Phi_{r}$) was about twice higher than for the membranes doped with PdTFPP (Φ = 2.8% in CHCl${}_{3}$ [40]). Even at the highest concentrations of O${}_{2}$, both optodes showed little amplitude excursion. The initial phosphorescence intensities for 21% O${}_{2}$ and pure nitrogen gas ([O${}_{2}$] < 0.0001%) varied by approximately 20% for IrTFPP-CO-Cl and 25% for PdTFPP.

The width of the excitation pulse permits modulation of phosphorescence intensity. Signal frequency should provide complete extinction of the emission between one detection cycle and the next (the time between pulses should be at least four-times the longest decay time) [35,41]. However, the lower its value, the greater the time required to process the respective lifetimes. In addition to the small number of averages used, no more than 1/3 of the maximum output current of the photomultiplier tube (100 μA) was required for the utilized excitation condition. As a result, the device developed permits even greater emission intensities than those obtained, and that would allow the higher repeatability measurements.

#### 3.3.2. Phosphorescence Lifetimes

Table 2 shows the values of the measured phosphorescence lifetimes for both optodes and methods (Equations (2) and (3)), the goodness of fit (r${}^{2}$) for the acquired curves and their respective uncertainties (three-times the standard deviation). The lifetimes experienced a typical descending hyperbolic variation as a function of the linear increase in the concentration of the quenching gas [7]. The nitrogen gas decays were seen to be exclusively monoexponential. As for concentrations with O${}_{2}$, decays presented a higher correlation with double exponential (r${}^{2}$ > 0.9998) [42,43]. However, unless the absolute times of the phenomenon are being defined, the method suggested by Equation (3) can be applied for the optodes, as well as for the instrument developed, since lifetimes are obtained with suitable repeatability.

**Figure 7:**
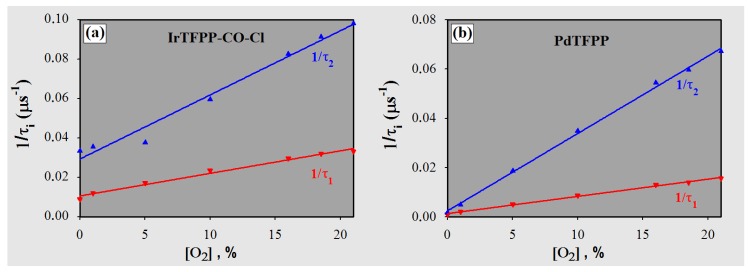
Reciprocals of $\tau_{1}$ and $\tau_{2}$ versus [O${}_{2}$] for (**a**) IrTFPP-CO-Cl and (**b**) PdTFPP immobilized in the polystyrene.

The appearance of multi- or even non-exponential luminescence decays is related to the inhomogeneous nature of the sensing membrane, in other words, the different microenvironments that exist within a polymer matrix. The main reasons for this heterogeneity include micro- and nano-crystallization of indicator (which provides islands that are less sensitive to quenching) [44,45], the distribution of distances between the probe molecules and the interacting parts of the polymer (i.e., spatial disorder that leads to a distribution of lifetimes) [42,46], as well as the possible existence of empty spaces within the host matrix [10]. When two lifetimes (τ longs and shorts) are used to approximate the decay profile of a monomodal distribution of lifetimes, each one of them should produce a linear Stern–Volmer plot ($\tau_{0}$/τ× [O${}_{2}$] or 1/τ× [O${}_{2}$]; see Section 3.3.3) [46]. Figure 7 shows the dependence of the reciprocals (1/τ) with the quencher concentration for both indicators embedded in polystyrene. As for the nature of the polymer matrix, the two membranes were lightly doped with poly(dimethylsiloxane-*co*-α-methyl styrene), which contributes to the heterogeneity of the medium. In the case of IrTFPP-CO-Cl/PS, the degree of non-linearity presented suggests that their complexity goes beyond a possible symmetrical monomodal or bimodal distribution of lifetimes [46]. The presence of aggregations of the indicator, eventual penta-coordinated derivatives in the sensor or even a greater spatial disorder should not be discarded. The analysis of phosphorescence decays alone is not sufficient to further investigate the nature of this microheterogeneity, and some authors have suggested other methods like fluorescence microscopy [44,47], as well as X-ray diffraction [46].

In systems where the magnitude of excitation allows the concentration of molecules in the excited states to remain constant (which is seen as a plateau before decay), intensities and lifetimes ratios are ideally close (e.g., I${}_{0}$/I${}_{21\%}$ = $\tau_{0}$/$\tau_{21\%}$). However, as seen, if the incident energy on the optodes is relatively low, initial phosphorescence intensities do not suffer the same attenuation as lifetimes (Figure 6) [48]. In this way, using high-sensitivity photodetectors makes the use of indicators with longer times (such as PdPs) more feasible, even in polymers with moderate permeability (such as PS) [7]. In summary, the approach employed with the home-built instrument extends the optode’s detection limit, since lifetimes can be accurately determined due to the preservation of the brightness parameter (maintenance of the SNR).

#### 3.3.3. Stern–Volmer Plots

For collisional quenching, the relationship between phosphorescence lifetimes and quencher concentration is expressed by the Stern–Volmer equation [3,12,18]:

(4) $$\frac{\tau_{0}}{\tau }=1+k_{q}.\tau_{0}.\left[O_{2}\right]=1+K_{SV}.\left[O_{2}\right],$$

where $\tau_{0}$ and τ: phosphorescence lifetimes in the absence and presence of O${}_{2}$, respectively; *k${}_{q}$*: bimolecular quenching constant; and *k*${}_{q}$.$\tau_{0}$ = *K*${}_{SV}$: Stern–Volmer constant.

Normally, a strictly linear dependence can be observed when the indicators are dissolved in a liquid solvent (quencher and dye diffusions remain the same throughout the sample). In such cases, phosphorescence decays can be modeled by a monoexponential [46]. However, since typical response curves for solid optical oxygen probes usually curve slightly downward [10,18,23,42,47], a derived version of the Stern–Volmer equation that accounts for this non-linear behavior has been applied (i.e., a model consistent with multi-exponential fitting of lifetimes). Thus, the two-site model was used and corroborates the lifetimes estimated by Equation (2) [7,10,37,49,50,51]:

(5) $$\frac{<\tau_{0}>}{<\tau >}={\left[\frac{f_{1}}{1+K_{SV_{1}}.\left[O_{2}\right]}+\frac{f_{2}}{1+K_{SV_{2}}.\left[O_{2}\right]}\right]}^{-1},$$

where <$\tau_{0}$> and <τ>: phosphorescence amplitude-weighted lifetimes in the absence and presence of O${}_{2}$, respectively; *f*${}_{1}$ and *f*${}_{2}$: fractions of the total emission for each component (with *f*${}_{1}$ plus *f*${}_{2}$ being one); and *K*${}_{SV_{1}}$ and *K*${}_{SV_{2}}$: Stern–Volmer constants. It is important to note that with the developed instrument, the stationary regime is not reached for phosphorescence intensities. Thus, the terms *f*${}_{1}$ and *f*${}_{2}$ of Equation (5) are the initial phosphorescence intensities of each one of the decay profiles (transient intensities).

On the other hand, lifetimes calculated by Equation (3) were used with a simplified heterogeneous model. This model considers the existence of two predominant localities/instances in the sensing membrane, one of which is virtually immune to quenching (Lehrer’s model) [18,22,39,42]:

(6) $$\frac{\tau_{0}}{\tau }={\left[\frac{f}{1+K_{SV}.\left[O_{2}\right]}+\left(1-f\right)\right]}^{-1},$$

where *f*: fractional contribution of the emission of the dominant instance; *K*${}_{SV}$: Stern–Volmer constant for the instance susceptible to quenching; and (1 − *f*): contribution of emission from the second instance, for which *K*${}_{SV}$ is considered null.

The use of Equation (6) is quite common in the systems that measure lifetimes by the phase shift and the modulation techniques [18,22,39] (in fact, for multi-exponential or non-exponential decays, this technique only gives apparent lifetimes that represent a complex weighted average of the decay components) [22,35]. More importantly, Equation (6) has significant practical application, since it allows calibration of non-linear devices from two points [9,39].

**Figure 8:**
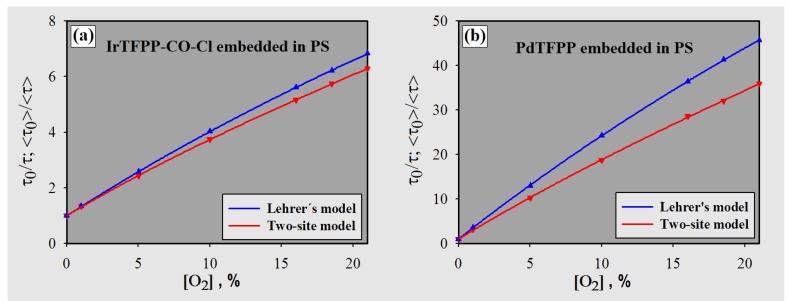
Modified Stern–Volmer plots for (**a**) IrTFPP-CO-Cl and (**b**) PdTFPP embedded in polystyrene (r${}^{2}$ > 0.9999 for all cases).

Equations (5) and (6) were employed to obtain the parameters summarized in Table 3 and to plot the calibration curves (solid lines) shown in Figure 8. According to Equation (4), *K${}_{SV}$* depends on *kq* and $\tau_{0}$. The *kq* values are numerically close (rigid media), since they depend on the collisional efficiency of the phosphorescence process and mainly on the diffusion coefficient of O${}_{2}$ in polystyrene (accessibility to the quencher) [7,52]. As for the indicators, the metal cation allows the magnitude of the dynamic quenching process to be adjusted, in other words, adjustment of the exposure window for the excited state of the metalloporphyrin to the molecular oxygen (e.g., Pd(II) > Rh(III) > Pt(II), considering metalation of TPP(p-CO${}_{2}$CH${}_{3}$)${}_{4}$) [3,8,20,48].

The curves of Figure 8 present a slightly downward curvature. It should be noted that the use of Equation (4) with the measured lifetimes (data presented in Table 2) results in an almost straight line (r${}^{2}$ > 0.9974). This can be considered linear; however, for lower concentrations of O${}_{2}$, the deviation between lifetime ratios and corresponding points in the calibration curve is quite significant: ≈15% for PdTFPP ([O${}_{2}$] = 1.01%, double exponential approach) and ≈6% for the iridium complex ([O${}_{2}$] = 5.01%, mono and double exponential approach). These differences mean less accuracy to detect O${}_{2}$. Therefore, the models used in this work (Equations (5) and (6)) aim to reduce such errors. In fact, they allow better determination of O${}_{2}$ concentrations with the measured lifetimes by means of the calibration curves. Table 4 shows the measurement errors calculated with the curves obtained for both models. The maximum errors were measured as 6.5% and 1.9% (two site-model) for [O${}_{2}$] = 1.01%, considering PdTFPP and IrTFPP-CO-Cl, respectively.

As seen in Figure 8, PdTFPP in PS presented a larger dynamic range than IrTFPP-CO-Cl (for the parameter Δτ/Δ[O${}_{2}$]), even considering higher concentrations (depending on the technique used and/or the presence of luminophore microdomains with different accessibilities, the sensitivity parameter is subject to saturation) [28]. In fact, indicators with long lifetimes offer higher resolution, but usually require the use of low-permeability polymers [3,7]. Iridium metalloporphyrin has presented a significantly greater magnitude of phosphorescence and a lower sensitivity (about six-times) than its palladium counterpart. However, because of the methodology used and despite the detection range considered, the Ir(III) complex was not shown to be more suitable; in other words, as seen in Table 2, a significant difference in repeatability (3σ) was not observed for higher quencher concentrations. In the specific case of the IrTFPP-CO-Cl indicator, its sensitivity is approximately 35% lower than chloro-(octaethylporphyrinato)-carbonyl iridium(III) (Ir-OEP-CO-Cl) embedded in PS as obtained by other authors [25], which is in accordance with the difference between the TFPP and OEP porphyrins (generally obtained in metalloporphyrin oxygen probes) [3,7].

### 3.4. Photostability

**Figure 9:**
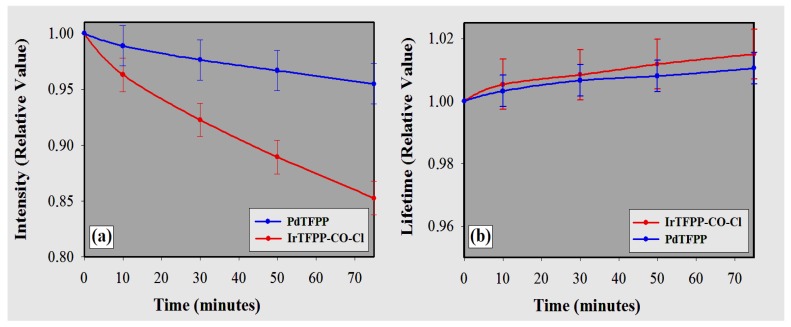
Measured phosphorescence (photobleaching) of polystyrene O${}_{2}$ optodes at 405 nm (continuous illumination, power dissipation of LED ≈ 68 mW). The values of the vertical axes are relative to the initial values (referenced as one) for both (**a**) intensity and (**b**) lifetime.

Figure 9 presents the results for the photostability tests. Voltage amplitudes (intensities) and lifetimes percentages were obtained in relation to the initial measurements ([O${}_{2}$] = ambient air; the error bars correspond to the maximum uncertainty observed in six optodes from the same batch: PdTFPP with ±0.50% for lifetimes and ±1.80% for intensities, while IrTFPP-CO-Cl ±0.80% and ±1.50%, respectively).

The pulsed-source time-resolved phosphorimetry method allows the use of low levels of excitation energy. Compared to phosphorescence intensity method, this requires a potentially more intense excitation beam, since the steady state of the primary sensor must be reached in order to obtain enough sensitivity (sufficient variation in intensities ratios). The detection phase method (frequency-domain), in turn, usually uses sinusoidally-modulated excitation light, constantly emitting energy [12,14,16]. As for the sensing phase, the compatibility of solubility (the similarity of the polarity between the metalloporphyrin and the host matrix) is a determining factor in the final stability of the optode [4,53]. The polystyrene itself is prone to photodegradation, but much more slowly than the indicators [50]. In theory, violet excitation of the IrTFPP-CO-Cl could generate chloride radicals that would decompose the metalloporphyrin [54]. In addition, also the release of the CO ligand could lead to a less stable five-coordinate complex; thus, both effects contribute to the lower stability of the Ir(III) complex.

Aside from the optodes investigated, little variation was seen in the calibration point for the transduction method based on phosphorescence lifetime. Although lifetimes undergo less variation than losses of amplitude, they cannot be regarded as stable enough to ignore photobleaching. However, they require system calibration much less frequently. The membranes were seen to lose more intense brightness initially, since the photodegradation rates decreased over time. Halogenated porphyrins generally present better performance in terms of robustness [3,21,22,24], but lifetime detection cannot always be considered a final solution to overcome the photodecomposition of a probe [49], since the drift of the decay time may also be significant according to the excitation energy and the compound employed (for example, cyclometalated iridium(III) coumarin complexes [50] and PdTPTBPF [49]).

If the membranes were ideally homogeneous, only differences relative to the initial phosphorescence intensities would be detected (reduction of the luminophore population). However, the sensing phase has multiple instances at which there is sensitivity to quenching, and also photostability, depending on the concentration of the indicator and the ease of access to the O${}_{2}$ in situ [55,56], justifying possible variation in lifetimes linked to intensities. In fact, reducing the width of the excitation pulse from 5 μs to 4 μs leads to a decrease of approximately 15% in intensities in ambient air, resulting in the reduction of the respective lifetimes of about 1.0%.

## 4. Conclusions

An instrument tuned to the spectral properties of the probes was designed specifically for characterization of O${}_{2}$ optodes. It is possible to change several characteristics of the measuring system by altering relatively inexpensive components like LED sources and optical filters and then matching the optical properties of these components with other dyes and polymers, resulting in a versatile instrument (open and low cost measurement system). The sensing methodology applied combined pulsed-source time-resolved phosphorimetry with a high-sensitivity photodetector. Despite the intense degree of dynamic suppression presented by the palladium metalloporphyrin probe, the low attenuation of the phosphorescence intensities makes possible lifetimes’ measurements with good repeatability for both polystyrene optodes, even for O${}_{2}$ concentrations close to ambient air. Therefore, the technique has not only allowed the description of different O${}_{2}$ optodes, but it can also offer potential benefits: expanded upper detection limits without significant limitation of the dynamic response and larger dynamic range compared to lower-sensitivity indicators. Under regular operating conditions for this system, the photodegradation of the O${}_{2}$ transducers was not noticeable, even during several hours of use. As a result, an additional advantage of this technique lies in the low excitation energy applied to the optodes, which is particularly useful in applications that require high photostability (e.g., continuous long-term monitoring of respiratory gaseous oxygen up to and around 21%).

Oxygen optodes based on the immobilization of IrTFPP-CO-Cl and PdTFPP in PS were investigated specifically. The metalation of TFPP with Ir(III), in comparison with Pd(II), caused a bathochromic shift of approximately 7 nm in the absorption and emission spectra. Both transducers showed good phosphorescence at ambient temperature (23 ${}^{\circ }$C); when IrTFPP-CO-Cl was embedded into PS, it presented significantly higher emission intensity than PdTFPP. Lifetimes for O${}_{2}$ concentrations ranging from 0–21% were investigated, indicating more complex dynamics when O${}_{2}$ is present. $\tau_{0}$ was measured as approximately 100 μs (IrTFPP-CO-Cl) and 1000 μs (PdTFPP). Regardless of the method used for data analysis (double or single exponential), the results have shown great reproducibility, and the modified Stern–Volmer plots fit the experimental data accurately. The application of Lehrer’s model has provided a simplified version to obtain calibration curves, as well as less reading errors for both O${}_{2}$ sensors. Ratios for lifetimes ($\tau_{0}$/$\tau_{21\%}$) were determined to be 6.3 and 6.8, and 35.9 and 45.6 for Ir(III) and Pd(II) complexes, respectively (two-site and Lehrer models, respectively). The same parameter for the Pt(II) complex in PS was obtained by other authors [57,58] and is <3, with $\tau_{0}$ about 60 μs. As for photostability, the robust PdTFPP indicator was seen to be better than its Ir(III) counterpart, despite its good performance (phosphorescence intensity decreased to 5% for the Pd(II) complex and 15% for the Ir(III) analogue after 75 min of continuous irradiation). Therefore, IrTFPP-CO-Cl immobilized in PS presents a promising alternative for O${}_{2}$ detection, offering potentially mid-range sensitivity over Pt(II) and Pd(II) metalloporphyrins.

## Figures and Tables

**Table 1:** Absorption peak wavelength ($\lambda^{abs}$), molar absorptivity (ε), emission peak wavelength ($\lambda^{em}$) and relative quantum yield ($\Phi_{r}$) of the indicators in chloroform and polystyrene films.

Indicator	$\lambda^{\mathrm{abs}}$nm (ε) M${}^{-1}$ cm${}^{-1}$ ${}^{a}$		$\lambda^{\mathrm{em}}$nm ${}^{b}$	$\Phi_{r}\;$ ${}^{c}$
	B Band	Q Band		
PdTFPP	407 (250,000)	520 (22,800), 553 (20,200)	673	1.0
IrTFPP-CO-Cl	414 (195,000)	527 (19,200), 558 (6100)	681	2.3

${}^{a}$ chloroform; ${}^{b}$ polystyrene; ${}^{c}$ $\Phi_{r}$ for IrTFPP-CO-Cl in PS was estimated relative to the PdTFPP counterpart ($\Phi_{r}$ = 1); uncertainties: ±6% for ε, ±2 nm for λ, and ±15% for $\Phi_{r}$.

**Table 2:** Measured lifetimes ${}^{a}$, coefficients of determination (r${}^{2}$ ${}^{b}$) and uncertainties (3σ ${}^{c}$) for the indicators in polystyrene.

**[O_2_] (%)**	**IrTFPP-CO-Cl**				
	**<τ> (μs)**	**r${}^{2}$**	**τ (μs)**	**r${}^{2}$**	**3σ (%)**
0.00	102.8	0.9999	103.1	0.9997	0.36
1.01	77.19	0.9999	77.02	0.9997	0.38
5.01	42.27	0.9999	39.91	0.9995	0.57
9.99	27.46	0.9999	25.62	0.9991	0.72
16.00	19.91	0.9999	18.39	0.9989	0.87
18.51	17.89	0.9999	16.57	0.9988	0.94
21.00	16.35	0.9999	15.09	0.9988	1.04
**[O${}_{2}$] (%)**	**PdTFPP**				
	**<τ> (μs)**	**r** ${}^{2}$	τ **(** μ **s)**	**r** ${}^{2}$	**3** σ **(%)**
0.00	1042	0.9999	1038	0.9999	0.27
1.01	327.5	0.9999	293.4	0.9991	0.28
5.01	101.2	0.9999	79.83	0.9980	0.53
9.99	55.54	0.9999	42.70	0.9975	0.66
16.00	36.47	0.9998	28.52	0.9974	0.85
18.51	32.55	0.9998	25.15	0.9974	1.01
21.00	29.01	0.9998	22.75	0.9972	1.04

${}^{a}$: <τ> and τ are phosphorescence lifetimes estimated from Equations (2) and (3), respectively; ${}^{b}$ coefficient of determination for the corresponding method; and ${}^{c}$ σ is the standard deviation calculated from 100 measurements.

**Table 3:** Parameters for polystyrene optodes using Equations (5) and (6) (two-site and Lehrer models).

Indicator	Two-Site Model					Lehrer’s Model		
	$f_{1}$	$f_{2}$	$K_{SV_{1}}$	$K_{SV_{2}}$	<$\tau_{0}$>/<τ>	*f*	$K_{SV}$	$\tau_{0}$/τ
IrTFPP-CO-Cl	0.9470	0.0530	0.325	0.0189	6.3	0.9734	0.339	6.8
PdTFPP	0.9932	0.0068	1.994	0.0218	35.9	0.9964	2.545	45.6

**Table 4:** Relative deviations between measured lifetimes ratios and calibration curves for PS optodes.

[O${}_{2}$] (%)	IrTFPP-CO-Cl		PdTFPP	
	$\Delta_{1}$(%) ${}^{a}$	$\Delta_{2}$(%) ${}^{b}$	$\Delta_{1}$(%) ${}^{a}$	$\Delta_{2}$(%) ${}^{b}$
0.00	+0.0	+0.0	+0.0	+0.0
1.01	+1.9	+0.6	+6.5	+0.1
5.01	−0.6	+0.1	−0.6	−1.1
9.99	+0.2	+0.1	−0.4	+0.3
16.00	−0.1	−0.1	+0.7	−0.2
18.51	+0.1	−0.2	−0.5	+0.1
21.00	−0.1	+0.1	+0.1	−0.2

^*a*^ considering the two-site model; and ^*b*^ Lehrer’s model.

## Abbreviations

The following abbreviations are used in this manuscript:

Ir-OEP-CO-Cl	Chloro(octaethylporphyrinato)-carbonyl iridium(III)
IrTFPP-CO-Cl	Chloro(*meso*-tetrakis(pentafluorophenyl)porphyrinato)-carbonyl iridium(III)
fwhm	Full width at half maximum
LabVIEW	Laboratory virtual instrument engineering workbench
LED	Light emitting diode
OEP	Octaethylporphyrin
OLEDs	Organic light emitting diodes
PdPs	Palladium porphyrins
PdTFPP	Palladium(II) *meso*-tetrakis(pentafluorophenyl)porphyrin
PdTPTBPF	Palladium(II) *meso*-tetrakis(4-fluorophenyl)tetrabenzoporphyrin
PMT	Photomultiplier tube
PS	Polystyrene
PtPs	Platinum porphyrins
PtTFPP	Platinum(II) *meso*-tetrakis(pentafluorophenyl)porphyrin
Ru(dpp)${}_{3}$ 2${}^{+}$	Ruthenium(II) diphenylphenanthroline
SNR	Signal-to-noise ratio
TFPP	*meso*-tetrakis(pentafluorophenyl)porphyrin
TIA	Transimpedance amplifier
TPP(p-CO${}_{2}$CH${}_{3}$)${}_{4}$	*meso*-tetra(*para*-methoxycarbonylphenyl)porphyrin
TPTBP	*meso*-tetra(phenyl)tetrabenzoporphyrin

## References

[1] Mathew S., Yella A., Gao P., Humphry-Baker R., Curchod B.F.E., Ashari-Astani N., Tavernelli I., Rothlisberger U., Nazeeruddin M.K., Gratzel M. (2014). Dye-sensitized solar cells with 13% efficiency achieved through the molecular engineering of porphyrin sensitizers. Nat. Chem..

[2] Sommer J.R., Shelton A.H., Parthasarathy A., Ghiviriga I., Reynolds J.R., Schanze K.S. (2011). Photophysical properties of near-infrared phosphorescent *π*-extended platinum porphyrins. Chem. Mater..

[3] Quaranta M., Borisov S., Klimant I. (2012). Indicators for optical oxygen sensors. Bioanal. Rev..

[4] Mills A. (1997). Optical oxygen sensors—Utilising the luminescence of platinum metals complexes. Platin. Met. Rev..

[5] Ni Y. (2008). Metalloporphyrins and functional analogues as MRI contrast agents. Curr. Med. Imaging Rev..

[6] Roussakis E., Li Z., Nowell N.H., Nichols A.J., Evans C.L. (2015). Bright, “clickable” porphyrins for the visualization of oxygenation under ambient light. Angew. Chem. Int. Ed..

[7] Wang X.D., Wolfbeis O. (2014). Optical methods for sensing and imaging oxygen: Materials, spectroscopies and applications. Chem. Soc. Rev..

[8] Rumyantseva V.D., Ivanovskaya N.P., Konovalenko L.I., Tsukanov S.V., Mironov A.F., Osin N.S. (2008). Synthesis and spectral luminescent characteristics of the porphyrin complexes with the platinum group metals. Rus. J. Bioorgan. Chem..

[9] Borisov S.M., Nuss G., Klimant I. (2008). Red light-excitable oxygen sensing materials based on platinum(II) and palladium(II) benzoporphyrins. Anal. Chem..

[10] Wolfbeis O. (2015). Luminescent sensing and imaging of oxygen: Fierce competition to the Clark electrode. Bioessays.

[11] Chu C.S., Lo Y.L. (2008). Ratiometric Fiber-optic oxygen sensors based on sol-gel matrix doped with metalloporphyrin and 7-amino-4-trifluoromethyl coumarin. Sens. Actuators B: Chem..

[12] Grist S.M., Chrostowski L., Cheung K.C. (2010). Optical oxygen sensors for applications in microfluidic cell culture. Sensors.

[13] Kostov Y., Rao G. (2000). Low-cost optical instrumentation for biomedical measurements. Rev. Sci. Instrum..

[14] Stich M.I.J., Fischer L.H., Wolfbeis O.S. (2010). Multiple fluorescent chemical sensing and imaging. Chem. Soc. Rev..

[15] Wang X.D., Chen H.X., Zhao Y., Chen X., Wang X.R. (2010). Optical oxygen sensors move towards colorimetric determination. Trends Anal. Chem..

[16] Rae B.R., Muir K.R., Gong Z., McKendry J., Girkin J.M., Gu E., Renshaw D., Dawson M.D., Henderson R.K. (2009). A CMOS time-resolved fluorescence lifetime analysis micro-system. Sensors.

[17] Mak C.S.K., Pentlehner D., Stich M., Wolfbeis O.S., Chan W.K., Yersin H. (2009). Exceptional oxygen sensing capabilities and triplet state properties of Ir(ppy-NPh_2_)_3_. Chem. Mater..

[18] Ast C., Schmalzlin E., Lohmannsroben H.G., Van Dongen J.T. (2012). Optical oxygen micro- and nanosensors for plant applications. Sensors.

[19] Ishihara S., Labuta J., Rossom W.V., Ishikawa D., Minami K., Hill J.P., Ariga K. (2014). Porphyrin-based sensor nanoarchitectonics in diverse physical detection modes. Phys. Chem. Chem. Phys..

[20] Capuano R., Pomarico G., Paolesse R., Di Natale C. (2015). Corroles-porphyrins: A teamwork for gas sensor arrays. Sensors.

[21] Borisov S.M., Papkovsky D.B., Ponomarev G.V., DeToma A.S., Saf R., Klimant I. (2009). Photophysical properties of the new phosphorescent platinum(II) and palladium(II) complexes of benzoporphyrins and chlorins. J. Photochem. Photobiol. A: Chem..

[22] Niedermair F., Borisov S.M., Zenkl G., Hofmann O.T., Weber H., Saf R., Klimant I. (2010). Tunable Phosphorescent NIR oxygen indicators based on mixed benzo- and naphthoporphyrin complexes. Inorgan. Chem..

[23] Wang X.D., Stolwijk J.A., Sperber M., Meier R.J., Wegener J., Wolfbeis O.S. (2013). Ultra-small, highly stable, and membrane-impermeable fluorescent nanosensors for oxygen. Methods Appl. Fluoresc..

[24] Enko B., Borisov S.M., Regensburger J., Baumler W., Gescheidt G., Klimant I. (2013). Singlet oxygen-induced photodegradation of the polymers and dyes in optical sensing materials and the effect of stabilizers on these processes. J. Phys. Chem. A.

[25] Koren K., Borisov S.M., Saf R., Klimant I. (2011). Strongly phosphorescent Ir(III)-porphyrins—New oxygen indicators with tuneable photophysical properties and functionalities. Eur. J. Inorg. Chem..

[26] Koren K., Dmitriev R.I., Borisov S.M., Papkovsky D.B., Klimant I. (2012). Complexes of Ir(III)-octaethylporphyrin with peptides as probes for sensing cellular O_2_. Chembiochem.

[27] Palmer J.H., Durrel A.C., Gross Z., Winkler J.R., Gray H.B. (2010). Near-IR Phosphorescence of iridium(III) corroles at ambient temperature. J. Am. Chem. Soc..

[28] Lebedev A.Y., Cheprakov A.V., Sakadzic S., Boas D.A., Wilson D.F., Vinogradov S.A. (2009). Dendritic phosphorescent probes for oxygen imaging in biological systems. ACS Appl. Mater. Interfaces.

[29] Hutter L.H., Muller B.J., Koren K., Borisov S.M., Klimant I. (2014). Robust optical oxygen sensors based on polymer-bound NIR-emitting platinum(II)-benzoporphyrins. J. Mater. Chem. C.

[30] Costa-Fernandez J.M., Bordel N., Campo J.C., Ferrero F.J., Perez M.A., Sanz-Medel A. (2000). Portable fibre optic oxygen sensor based on room-temperature phosphorescence lifetime. Microchim. Acta.

[31] Hamamatsu Photonics K K, Electron Tube Division. Photomultiplier Tubes Modules.

[32] Wang T., Erhman B. (2005). Compensate Transimpedance Amplifiers Intuitively.

[33] Idris A.C., Saad M.R., Zare-Behtash H., Kontis K. (2014). Luminescent measurement systems for the investigation of a scramjet inlet-isolator. Sensors.

[34] Lamprecht B., Tschepp A., Cajlakovic M., Sagmeister M., Ribitsch V., Kostler S. (2013). Luminescence lifetime-based capillary oxygen sensor utilizing monolithically integrated organic photodiodes. Analyst.

[35] Lakowicz J.R. (2006). Principles of Fluorescence Spectroscopy.

[36] Guerci P., Ince Y., Heeman P., Faber D., Ergin B., Ince C. (2017). A LED-based phosphorimeter for measurement of microcirculatory oxygen pressure. J. Appl. Phys..

[37] Banerjee S., Kuznetsova R.T., Papkovsky D.B. (2015). Solid-state oxygen sensors based on phosphorescent diiodo-borondipyrromethene dye. Sens. Actuators B: Chem..

[38] Ji S., Wu W., Wu Y., Zhao T., Zhou F., Yang Y., Zhang X., Liang X., Wu W., Chi L., Wang Z., Zhao J. (2009). Real-time monitoring of luminescent lifetime changes of PtOEP oxygen sensing film with LED/photodiode-based time-domain lifetime device. Analyst.

[39] Borisov S., Zenkl G., Klimant I. (2010). Phosphorescent platinum(II) and palladium(II) complexes with azatetrabenzoporphyrins - new red laser diode-compatible indicators for optical oxygen sensing. ACS Appl. Mater. Interfaces.

[40] To W.P., Liu Y., Lau T.C., Che C.M. (2013). A robust palladium(II)-porphyrin complex as catalyst for visible light induced oxidative C-H functionalization. Chem. A Eur. J..

[41] Leung R.W.K., Yeh S.C.A., Fang Q. (2011). Effects of incomplete decay in fluorescence lifetime estimation. Biomed. Opt. Express.

[42] Hartmann P., Trettnak W. (1996). Effects of polymer matrices on calibration functions of luminescent oxygen sensors based on porphyrin ketone complexes. Anal. Chem..

[43] Palma A.J., López-González J., Asensio L.J., Fernández-Ramos M.D., Capitán-Vallvey L.F. (2007). Microcontroller-based portable instrument for stabilised optical oxygen sensor. Sens. Actuators B: Chem..

[44] Kneas K.A., Demas J.N., DeGraff B.A., Periasamy A. (2000). Fluorescence microscopy study of heterogeneity in polymer-supported luminescence-based oxygen sensors. Microsc. Microanal..

[45] Payne S.J., Fiore G.L., Fraser C.L., Demas J.N. (2010). Luminescence oxygen sensor based on a ruthenium(II) star polymer complex. Anal. Chem..

[46] Hartmann P., Leiner M.J., Lippitsch M.E. (1995). Response characteristics of luminescent oxygen sensors. Sens. Actuators B: Chem..

[47] Kelly C.A., Toncelli C., Kerry J.P., Papkovsky D.B. (2014). Discrete O_2_ sensors produced by a spotting method on polyolefin fabric substrates. Sens. Actuators B: Chem..

[48] Valeur B., Berberan-Santos M.N. (2013). Molecular Fluorescence: Principles and Applications.

[49] Koren K., Hutter L., Enko B., Pein A., Borisov S.M., Klimant I. (2013). Tuning the dynamic range and sensitivity of optical oxygen-sensors by employing differently substituted polystyrene-derivatives. Sens. Actuators B: Chem..

[50] Borisov S.M., Klimant I. (2007). Ultrabright oxygen optodes based on cyclometalated iridium(III) Coumarin Complexes. Anal. Chem..

[51] Carraway E.R., Demas J.N., DeGraff B.A. (1991). Luminescence quenching mechanism for microheterogeneous systems. Anal. Chem..

[52] Borisov S.M., Nuss G., Haas W., Saf R., Schmuck M., Klimant I. (2009). New NIR-emitting complexes of platinum(II) and palladium(II) with fluorinated benzoporphyrins. J. Photochem. Photobiol. A: Chem..

[53] Woll D., Braeken E., Deres A., De Schryver F.C., Uji-i H., Hofkens J. (2009). Polymers and single molecule fluorescence spectroscopy, what can we learn?. Chem. Soc. Rev..

[54] Lemon C.M., Hwang S.J., Maher A.G., Powers D.C., Nocera D.G. (2018). Halogen photoelimination from SbV dihalide corroles. Inorg. Chem..

[55] Hartmann P., Leiner M.J.P., Kohlbacher P. (1998). Photobleaching of a ruthenium complex in polymers used for oxygen optodes and its inhibition by singlet oxygen quenchers. Sens. Actuators B: Chem..

[56] Xue R., Behera P., Xu J., Viapiano M.S., Lannutti J.J. (2014). Polydimethylsiloxane core–polycaprolactone shell nanofibers as biocompatible, real-time oxygen sensors. Sens. Actuators B: Chem..

[57] Koren K., Borisov S.M., Klimant I. (2012). Stable optical oxygen sensing materials based on click-coupling of fluorinated platinum(II) and palladium(II) porphyrins—A convenient way to eliminate dye migration and leaching. Sens. Actuators B: Chem..

[58] Basu B.J., Bharathida T., Rikhari B., Prasannan D., Kum V.D., Chakradhar R.P.S. (2012). Studies on the fabrication and characterization of optical sensor coatings for aerodynamic applications. J. Appl. Sci..

